# Giant parathyroid adenoma mimicking as gallstone disease and musculoskeletal disorders: a case report from a resource-limited setting

**DOI:** 10.1097/MS9.0000000000003764

**Published:** 2025-09-01

**Authors:** Sarada Khadka, Utsav Sitaula, Riya Thapa, Raju Basnet, Robin Maskey

**Affiliations:** a Department of Surgery, B.P. Koirala Institute of Health Sciences, Dharan, Nepal; b B.P. Koirala Institute of Health Sciences, Dharan, Nepal; c Department of Internal Medicine, B.P. Koirala Institute of Health Sciences, Dharan, Nepal

**Keywords:** case report, giant parathyroid adenoma, hypercalcemia, musculoskeletal pain, primary hyperparathyroidism

## Abstract

**Introduction and importance:**

Giant parathyroid adenomas (GPAs) are rare causes of primary hyperparathyroidism and may mimic various other disorders, leading to diagnostic delays. Early identification is key, especially in resource-limited settings.

**Presentation of case:**

We present the case of a 56-year-old woman with vague symptoms like body pain, constipation, and nocturia. She was initially misdiagnosed with gallstone disease and musculoskeletal pain. Further evaluation revealed a serum calcium of 16.04 mg/dl and a parathyroid hormone (PTH) level of 2118 pg/ml. Imaging confirmed a left parathyroid adenoma. Surgical excision of an 8.95 g mass led to resolution of symptoms and normalization of calcium and PTH.

**Clinical discussion:**

GPAs, though benign, often present with severe symptoms and biochemical abnormalities mimicking malignancy. Diagnostic challenges include overlapping presentations and limited imaging resources. Low urinary calcium in this patient was attributed to chronic renal adaptation.

**Conclusion:**

Giant parathyroid adenomas should be considered in patients with hypercalcemia and vague musculoskeletal symptoms. Timely diagnosis and surgery are curative, even in low-resource settings.

## Introduction

### Background

Parathyroid glands are small, oval masses located posterior to thyroid approximately 6 mm in length, 3–4 mm across, and 1–2 mm from posterior to anterior and weighing 50 mg which regulate calcium homeostasis via PTH secretion[1]. Parathyroid adenomas are benign tumors and account for the majority of primary hyperparathyroidism (PHPT) cases. Most adenomas weigh <1 g; those >3.5 g are defined as giant parathyroid adenomas (GPAs)^[2,3]^. A recent study showed that majority of patients with giant parathyroid adenoma had symptoms of fatigue, skeletal pain, pathological fracture, nausea and abdominal pain[4]. Surgical procedures have been standard treatment for giant parathyroid adenomas and recent study suggests focused parathyroid exploration has similar recurrence, persistence and reoperation rates with significantly lower complication rates with shorter operative time[5].

**HIGHLIGHTS**

- Giant parathyroid adenomas (>3.5 g) are rare and may mimic other common conditions.
- Misdiagnosis in this case was due to overlapping symptoms with gallstone disease and musculoskeletal disorders.
- Diagnosis was confirmed through PTH levels and imaging despite limited resources.
- Surgical excision led to full symptomatic and biochemical recovery.
- Emphasizes the need for broader differential diagnosis in persistent hypercalcemia.

### Rationale

GPA is rare and often misdiagnosed due to overlapping of its symptoms with that of musculoskeletal origin like skeletal pain, pathological fracture and treated otherwise. This case is noteworthy due to the extreme PTH elevation with elevated calcium levels, diagnostic delay, and overlap with gallstone and musculoskeletal symptoms in a resource-constrained setting.

### Literature and guidelines

Typical diagnostic methods include serum calcium, PTH assays, and imaging such as USG, sestamibi SPECT, or 4D CT[6]. However, in resource limited settings, limited access to advanced diagnostics can delay intervention.

### Guideline citation

This case report has been reported in line with the SCARE 2025 checklist[7].

### Patient information

A 56-year-old female presented to the outpatient department of an academic institution with a 5-year history of generalized body pain that had significantly worsened over the past 6 months (Table 1). She complained of generalized body pain most pronounced over the joints and back worsening with movement, severe enough to prevent her from moving from bed. Besides, her symptoms included nocturia, constipation occurring every 2–3 days, insomnia, nausea, and occasional vomiting. Initially, these complaints were attributed to gallstone disease and musculoskeletal discomfort, leading to symptomatic management with analgesics for pain without definitive resolution. Cholelithiasis was confirmed via imaging findings following which she was referred for laparoscopic cholecystectomy. In her preoperative workup, she was found to have hypercalcemia for which further workup were done. She had undergone a total abdominal hysterectomy 11 years prior and was recently diagnosed with cholelithiasis. She reported intermittent use of calcium and vitamin D supplements previously but there was no documents available and had no known drug allergies. There was no history of tobacco, alcohol, or recreational drug use. Her family history was non-contributory with no known endocrine or renal disorders. She lived with her family and remained independent in her daily activities. Notably, she denied any visual disturbances, headaches, or palpitations, and the body pain was most pronounced over the joints and back.

**Table 1:** Timeline of progression and management of the condition

Date	Events
2020	Onset of generalized of generalized body pain
2020–2023	The patient had generalized body pain throughout the time but of mild intensity for which she didn’t visit any medical facility.
2023	Started taking calcium and vitamin D tablets
December 2024	Bone pain worsened
16 April 2025	Visited surgery OPD for checkup and Gall stone was diagnosed from serum tests and USG findings and was planned for Laparoscopic cholecystectomy
	As total calcium was also raised, Got referred to medicine
17 April 2025	Admitted to medicine ward for diagnostic workup and medical management of hypercalcemia
18 April 2025	PTH was 932 pg/ml, total calcium was 16.04 mg/dl
20 April 2025	Hypercalcemia was medically managed and the increased in serum creatinine level was also noted and managed
21 April 2025	USG showed solid cystic lesion posterior to thyroid gland suggestive of mass of thyroid or parathyroid origin.
22 April 2025	CT neck and chest suggested parathyroid adenoma with cystic degeneration
24 April 2025	Referred to surgery and planned for parathyroidectomy under general anesthesia
28 April 2025	Pre-operative PTH was 2118 pg/ml
	Left focus superior parathyroidectomy was performed
	Postoperatively PTH dropped to 108 pg/ml
29 April 2025	PTH dropped to 19.4 pg/ml
	Total calcium reached a level of 9.25 mg/dl

### Clinical findings

Upon examination, the patient was hemodynamically stable with vital signs within normal limits (blood pressure: 100/80 mm Hg, pulse rate: 72 beats per minute, respiratory rate: 18 breathes/ min, and temperature: 98 degree Fahrenheit). She weighed 61 kg and had normal built. No palpable neck mass or lymphadenopathy was appreciated on physical examination. Abdominal and musculoskeletal system assessments revealed only diffuse tenderness without focal findings. Other systemic and physical examination findings were normal.

### Diagnostic assessment

**Figure 1.**
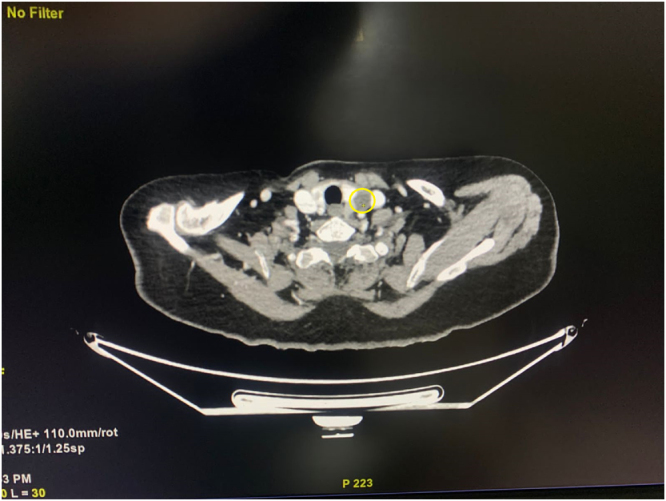
CECT neck in arterial phase showing a well-defined solid cystic lesion with enhancing solid component noted posterolateral to left thyroid gland; abutting thyroid lamina; laterally abutting left common carotid artery and left internal jugular vein likely to be parathyroid adenoma with cystic degeneration.

Initial laboratory evaluation revealed a profoundly elevated parathyroid hormone (PTH) level of 932 pg/ml and a markedly elevated serum calcium concentration of 16.04 mg/dl. The 24-hour urinary calcium was reduced at 61.71 mg, suggesting altered calcium handling with calcium creatinine ratio of 0.021. Renal function was mildly impaired with a serum creatinine level of 1.93 mg/dl. Liver enzymes including ALP, ALT, and AST were elevated, with ALP reaching 343.47 U/L, ALT 87.88 U/L, and ALP 90.75 U/L and a total calcium of 16.04 mg/dl (Table 2). Neck ultrasonography demonstrated a solid-cystic lesion posterior to the left thyroid lobe. Further localization with contrast-enhanced computed tomography (CECT) revealed a 2.6 × 2.4 × 3.6 cm mass adjacent to the left common carotid artery and jugular vein, consistent with a parathyroid adenoma (Fig. 1). Due to resource constraints, advanced imaging modalities such as sestamibi scans or 4D CT were not available. The overlapping symptoms with gallstone disease and musculoskeletal conditions contributed to a delay in reaching the correct diagnosis. However, the persistence of symptoms alongside worsening hypercalcemia prompted further endocrinological evaluation, leading to the diagnosis of primary hyperparathyroidism (PHPT).

**Table 2:** Laboratory investigations findings

Parameters	Result		Unit	Hospital ref. range
	Preoperative	Postoperative		
Serum PTH	2118 (pre-excision value)	19.4 (day 1)	pg/ml	6–80
Serum total calcium	16.04	9.25	mg/dl	8.4–10.2
ALT(SGPT)	90.75	–	U/L	09–43
AST(SGOT)	87.88	–	U/L	10–35
ALP	343.47	–	U/L	35–130
Serum creatinine	1.93	–	mg/dl	0.3–1.2
Urinary calcium	61.71	–	mg/24 hrs	100–321
Total urine protein	0.36	–	g/24 hrs	<0.15
24 hrs urine volume	3500	–	ml	1500–2500
Calcium creatinine clearance ratio	0.021	-	-	0.01–0.02

### Therapeutic intervention

Initially, after finding out hypercalcemia in preoperative workup, she was treated for hypercalcemia medically by giving her to drink 4 liters of water with furosemide 20 mg intravenous twice a day before recognizing the presence of parathyroid adenoma. After being diagnosed with parathyroid adenoma she was planned for surgery. Preoperatively, the patient was stabilized with correction of dehydration and electrolyte imbalances and kept nil per oral (NPO). Just before surgical procedure, her PTH was 2118 pg/ml .Following informed consent, a focused superior parathyroidectomy was performed on the left side.

Initially painting and draping was done. Ultrasound of neck was performed and parathyroid adenoma was localized and 4 cm incision was marked above the adenoma. Pre-incision value of PTH was sent and a left lateral incision was made. A subplatysmal flap was elevated, and a space was created between the sternocleidomastoid and strap muscles. The left jugular vein and carotid artery were identified and lateralized. The left superior parathyroid adenoma was identified and dissected, and its pedicular vessels were identified and ligated. Parathyroid hormone was sent 10 minutes after excision of parathyroid adenoma. Hemostasis was secured, fascia of strap muscle was approximated with fascia of sternocleidomastoid. Subcutaneos tissue was approximated with 3/0 vicryl interrupted sutures and skin closed with 3/0 monoglide suture and a sterile dressing was applied. The procedure was performed by an MCh-trained Breast, Endocrine, and General Surgeon with over 5 years of independent surgical experience, routinely managing complex thyroid and parathyroid cases in a tertiary care center. Standard surgical principles and institutional protocols were strictly followed. There was no any deviation from the planned intervention.

**Figure 2.**
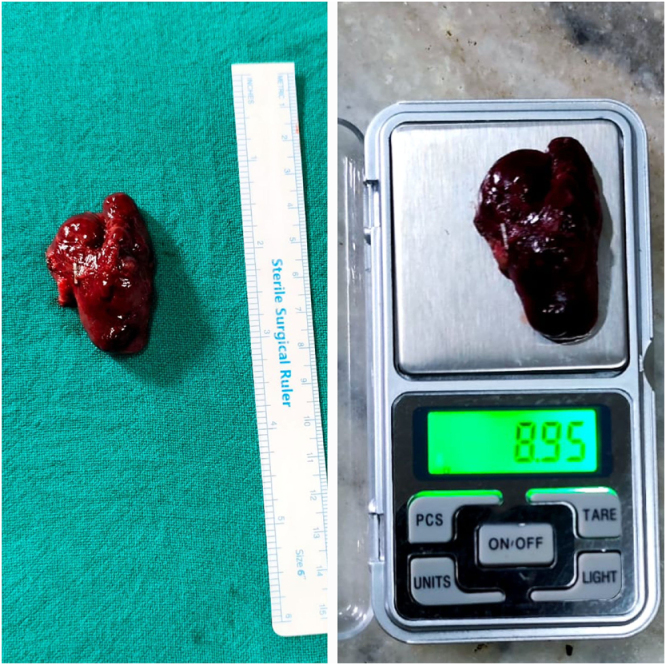
Surgically resected single globular mass grey white to blackish measuring 3.5×3×1.5 cm (left) and weighing 8.95 gram (right), which was encapsulated and no invasion to adjacent structures were noted.

**Figure 3.**
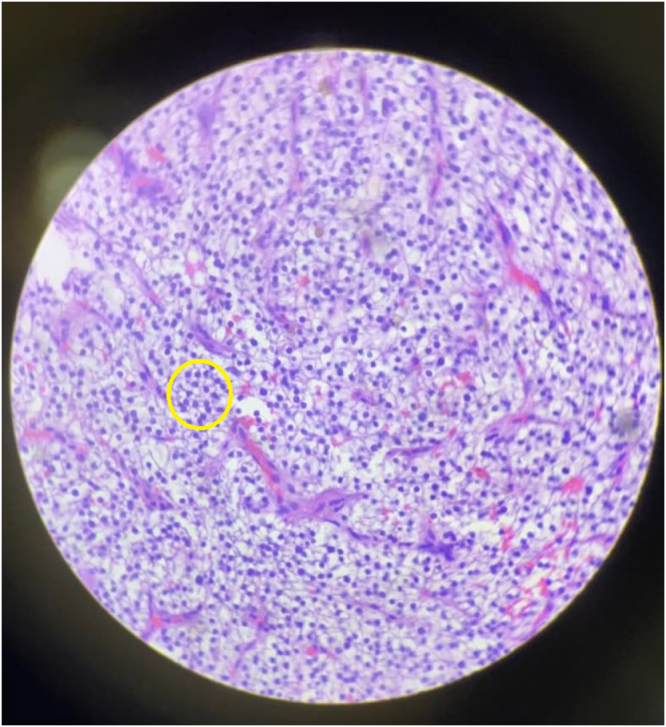
Histological image comprising of predominant chief cells arranged in diffuse sheets separated by delicate vasculature and few follicles containing colloid like material with no capsular and vascular invasion.

The excised mass measured 3.5 × 3 × 1.5 cm and weighed 8.95 grams (Fig. 2). Histopathological examination confirmed the diagnosis of a parathyroid adenoma with a partly capsulated lesion, comprising of predominantly chief cells arranged in diffuse sheets with no capsular and vascular invasion (Fig. 3). The patient resumed a liquid diet within 24 hours. Her PTH levels showed a rapid decline, dropping to 10 8 pg/ml (post-excision value) and normalizing to 19.4 pg/ml by the first post-operative day.

### Outcomes and follow-up

The postoperative course was smooth with no any postoperative complications. There was complete resolution of symptoms and normalization of serum calcium and PTH levels. Postoperatively, she was monitored for calcium levels for 5 consecutive days and after she was found to have normal calcium levels, she was discharged with calcium 500 mg four times a day and alpha d3 capsule 0.5 microgram twice a day. On 1 week and 1 month follow-up she is asymptomatic with normal PTH and calcium level. Long-term follow-up is pending and is planned for 3 month and 6 month visit with original operating surgeon in person for monitoring the patient and recurrence, but the short-term prognosis appears excellent with no evidence of recurrence or complications. The patient was adherent to the given advice and had been on regular follow-up.

## Discussion

Giant parathyroid adenomas, though histologically benign, often present with severe symptoms that can mimic parathyroid carcinoma due to their size and biochemical profile[8]. Studies states that preoperative diagnosis of parathyroid carcinoma is difficult before surgery but different clinical clues can be helpful like early age of presentation, markedly elevation of serum parathyroid hormone and a serum calcium of more than 14 mg/dl[9]. Parathyroid carcinoma was also kept in suspicion but it was ruled out post-report of histopathology came and with normalization of the calcium and parathyroid hormone levels post-surgery. Intraoperative assessment was made on the appearance, adherence and invasiveness of gland to surrounding structures to rule out parathyroid carcinoma with monitoring of intraoperative PTH level. However, a frozen section was not performed due to institutional limitations. In this case, the delayed diagnosis was largely due to initial presentation mimicking gallstone disease and musculoskeletal pain with concurrent comorbidities that diverted initial clinical suspicion. The patent was initially prescribed some calcium tablets previously in other hospitals in view of bone pain which is common in this age group due to osteoporosis[10].

This case gives us the idea that not all chronic bone pain are due to osteopenia or osteoporosis, something like parathyroid adenoma should also be kept in mind. Interestingly, despite severe hypercalcemia, the patient had low urinary calcium levels of 61.71 mg in 24 hours, possibly indicating renal adaptation in the context of chronic PHPT. This may raise concern for familial hypocalciuric hypercalcemia but no workup was required for low calcium in sporadic PHPT.[11] The comparison of the case with various similar cases has been made with all the cases having uneventful recovery^[12–17]^ (Table 3). This case emphasizes the importance of considering hyperparathyroidism in the differential diagnosis of chronic, unexplained musculoskeletal complaints and hypercalcemia, especially in resource-limited settings. Despite the lack of access to nuclear imaging, basic imaging modalities like ultrasonography and CECT proved sufficient for diagnosis and surgical planning.

**Table 3:** Comparison of various studies on giant parathyroid adenoma

S. No.	Authors & year	Presenting Symptoms	Serum Ca^2+^ (mg/dl)	PTH (pg/ml)	Size (cm)	Weight (g)	Imaging used	Pre-op diagnosis	Post-op outcome
1	Adina Ghemigian *et al* (Review)	Bone pain, fatigue, nephrolithiasis, GI symptoms	Elevated in giant adenomas significantly higher than in non‑GPA cases (no single value given)	Pre‑operative PTH levels are higher in GPA compared to typical small adenomas but no single typical numeric value is provided	-	-	USG, Sestamibi, CT	PHPT/GPA	Good outcomes in most
2	Siddharth Shah *et al* (2021)	Inability to work bilateral knee pain constipation frontal headache low back pain	>2000 pg/ml	>2000 pg/ml	3.2×2.2×1.5 cm	5.3	USG, Parathyroid nuclear scan	PHPT	Uneventful recovery
3	Rahim Mahmodlou *et al* (2018)	Knee pain, lower back pain, fatigue, and dizziness	14.6	2702	9×6×4cm.	122	Color Doppler ultrasonography, CT, 99mTc-MIBI scintigraphy	PHPT	Recovered well
4	Dildar Haji Musa *et al* 2023	Bone pain, fatigue, constipation, recurrent renal stones	16.5	1550	6.0×5.5×1	30	USG, MRI	PHPT vs carcinoma	Normalized Ca and PTH
5	Junwei Weng *et al* (2021)	Admitted to hospital with a 1-month history of hypercalcemia during arthroplasty, no other symptoms	12.52	413	4.3×3.2×6.3	-	USG,CT, SPECT IMAGING	PHPT	Resolved symptoms
6	Junwei Weng *et al* (2021)	Admitted to hospital following RTA, developed symptoms of obvious thirst, polyuria, decreased appetite, severe nausea, vomiting, and constipation	16.4	593.5	5.0×3.0×3.0	-	USG, CT, SPECT imaging	PHPT (Cystic GPA)	Resolved symptoms
7	Our case	Chronic pain, constipation, nausea	16.04	2118	3.5×3×1.5	8.95	USG, CECT	PHPT due to giant parathyroid adenoma	Complete recovery
8.	Giuseppe Evola *et al*	Persistent musculoskeletal pains, constipation, and polyuria for 2 years	12.5	2747	6.5×5.0×3.0 cm	90g	USG	Giant parathyroid adenoma	Normalized Ca and PTH
							CT Scan		

CECT, contrast enhanced computed tomography; CT, computed tomography; GI, gastrointestinal; MRI, magnetic resonance imaging; PHPT, primary hyperparathyroidism; PTH, parathyroid adenoma; RTA, road traffic accident; SPECT, single photon emission computed tomography; USG, ultrasonography.

### Strengths and limitations

A major strength of this case is its demonstration of successful diagnosis and management of a giant parathyroid adenoma using limited resources. It underscores the feasibility of curative surgery with the aid of essential but accessible diagnostic tools. However, advanced nuclear imaging and postoperative data of all preoperative tests were not available, and long-term follow-up data remain pending, representing limitations to comprehensive evaluation.

## Conclusion

This case reinforces the need to include giant parathyroid adenoma in the differential diagnosis of chronic body pain. Timely recognition and surgical intervention can lead to complete symptom resolution and biochemical normalization. Even in low-resource environments, a high index of suspicion combined with basic diagnostics can facilitate effective management.

## Data Availability

None.

## Research registration unique identifying number (UIN)

Open Science Framework (OSF) UIN: OSF-daju4 (https://osf.io/daju4/).

## Patient perspective

The patient reported significant relief and satisfaction following the surgical intervention. She expressed gratitude for the eventual diagnosis and treatment, having experienced years of discomfort and uncertainty regarding her condition. She said, “I am very thankful to the health professionals for helping me to recover from the pain and the problem I have been suffering for years.”
